# Biological assessment of the omega-3 status after omega-3 enriched dietary during an active seasonal performance on soccer players

**DOI:** 10.1186/s13102-024-00823-7

**Published:** 2024-03-22

**Authors:** Haeyoung Kim, Seungho Shin, In-ho Jeon, Jae-Man Kwak

**Affiliations:** 1 Department of Orthopedic Surgery, College of Medicine, Uijeongbu Eulji Medical Center, Eulji University, Uijeongbu, South Korea; 2 Wellcare Clinic, Seoul, South Korea; 3 Green grass bio. ltd, Seoul, Republic of Korea; 4 Department of Orthopedic Surgery, College of Medicine, Asan Medical Center, Ulsan University, Seoul, South Korea

**Keywords:** Omega-3, Omega enriched dietary, Soccer player, Trans fatty acid

## Abstract

**Background:**

To assess the physiological change of blood fatty acid composite during the seasonal performance of soccer players with omega-6/omega-3 polyunsaturated fatty acid balanced dietary supplementation.

**Methods:**

This study included 20 healthy and trained male soccer players. During the study, data collection was performed three times (pre-, mid-, post-season). Anthropometric data collection and blood sampling for the assessment of the omega index were performed. The mid- and post-seasonal data were compared with baseline data collected before the starting season (pre-seasonal data).

**Results:**

Elevated levels of omega-3, HS-omega, and trans fatty acid were observed in both the mid- and post-seasonal data. During the season, the levels of omega-6/omega-3 and saturated fatty acid decreased, whereas there were no differences in total cholesterol, cholesterol LDL, HDL, BUN/Cr, HbA1c (NGSP), and cystatin C.

**Conclusions:**

n-3 PUFA-enriched dietary supplementation might alter blood omega-3 indices in soccer players during the season.

## Introduction

Blood FA profiles are important for human health and can be influenced by dietary choices or supplementation [1]. Based on general perceptions and existing research, a diet rich in omega-3 fatty acids are commonly expected to improve heart health, reduce inflammation, and potentially benefit mental health and cognitive function [2]. Alpha-linolenic acid and its derivatives, namely, eicosapentaenoic acid (EPA) and docosahexaenoic acid (DHA), among other n-3 PUFAs, possess anti-inflammatory characteristics [3]. On the other hand, n-6 PUFAs, such as linoleic acid and its derivative, arachidonic acid (ARA), have proinflammatory effects [4]. Due to the opposing effect regarding inflammatory reaction, having a lot more omega-6 fatty acids compared to omega-3 fatty acids in the diet could significantly affect the body’s metabolism and inflammation in various ways [5, 6]. However, studies have been limited regarding whether these effects can also be expected in individuals such as athletes who engage in high-intensity exercises [7, 8].

Throughout the playing season, the majority of sportsmen, particularly soccer players, need to maintain the best possible muscular health. Several studies have investigated with nutritional perspective in preventing muscle injury from the perspective of an athlete’s training [9, 10]. The scholarly literature on the subject indicates that a number of chemicals and signaling cascades are involved in muscle health maintenance. By activating natural antioxidant enzymes, n-3 PUFA promotes protection against muscle injury and inflammation [11]^,^ [12]. The expression of genes that promote regeneration of injured skeletal muscles is particularly induced by an increase in inflammatory response [13].

Supplements to obtain n-3 PUFAs are present in marine sources, such as fatty fish (e.g., salmon, smelt, and anchovies), and fish oil supplements. However, these sources are underutilized owing to their unpleasant taste, accessibility issues, public concern regarding environmental toxins (e.g., mercury, polychlorinated biphenyls, chlordane, and dioxins), and vegetarian or vegan dietary preferences [14].

Thus, alternative sources of n-3 PUFAs are required for reasons other than consumer desire as the world’s finite supply of ocean fish cannot be sustained or scaled up to satisfy demand. Meat products enriched with dietary n-3 PUFA, such as omega-3 butter, beef, and oil, which are produced by cows fed with n-3 PUFA-rich feed, are being developed. These products have the advantages of a well-established infrastructure and strong consumer demand. The present study was conducted to investigate the blood fatty acid composition including omega index under daily dietary program using n-3 PUFA-enriched meat products and to evaluate its contribution to athletes in professional soccer league.

## Materials and methods

This study was approved by the research ethics committee of Asan Medical Center (IRB No. S2021-0153-0010). All methods were carried out in accordance with relevant guidelines and regulations. Informed consent was obtained from all participants.

### Participants

This study included 20 healthy and trained male soccer players. The team was part of the professional soccer league of Jeon-Nam, South Korea. The players’ mean age was 26.0 ± 3.79 years; mean body mass, 79.6 ± 6.99 kg; and mean height, 182.23 ± 7.28 cm. All players belonged to the soccer team that competed for the championship of Korean pro league that started on March 2021 and ended in December 2021. During the season (10 months), the athletes trained for approximately 3 h a day, 3 to 6 days a week, in the field, in addition to performing strength training three times a week for approximately 1 h. All athletes were housed at the team’s training center under the same conditions of accommodation, feeding, and routine schedule. None of the participants smoked or had taken any type of medication or dietary supplementation for at least 2 months prior to the start of the study. The daily dietary program including n-3 PUFA-enriched meat products was adopted 1 month before the start of the season.

### Diet preparation

Nutrient balance and composition conformed to the IOC consensus statement; dietary supplements and the high-performance athlete [15]. The recommended daily protein dose was 1.6-g protein/kg/day optimal (up to 2.2 g/kg/day with no adverse effects), and the recommended per-meal protein dose was 0.3–0.5-g protein/kg (3–4 times per day in close temporal proximity to exercise, with postexercise being consistently shown to be effective). The IOC consensus statement also recommended intake of omega-3 FAs (about 2 g/day for increasing muscle protein synthesis and reducing muscle-damaging exercise). Following IOC consensus for intake of omega-3 FAs, The n-3 PUFA-enriched meat products from omega-3–fed cows and pork were supplied by Green Grass (Chungju-si, Korea) and were commercially available (n-3 PUFA; 933–1067 mg/meal).

### Study design

The study lasted for one whole season period (10 months, Feb–Dec 2021). The present study was designed as the observational study to evaluate the differences in the same group of athletes but during different time-points of soccer season. During the study, data collection was performed three times (at 1 month before the season (baseline)), in the middle of the season (5 months after starting the season), and after the season (1 month after the last game was finalized). Anthropometric data collection and blood sampling were also performed. The athletes were instructed not to exercise during the weekend prior to the testing week. The coaching staff and researchers encouraged the athletes and questioned them regarding their supplementation and food intake per day during the study period.

### Nutritional and anthropometric evaluation

Body mass and height were measured using a scale and stadiometer. In addition, body mass index and fat/muscle/water composition were measured using a body composition analyzing system (InBody®, South Korea). The participants were instructed to follow their diet throughout the week and complete a food-recall form for 3 nonconsecutive days during the same week. The forms were analyzed to determine the total intake of calories, carbohydrates, proteins, lipids, and vitamins C, E, and A. Furthermore, the use of supplements was recorded and added to the food-recall form as part of habitual intake. During the study period, intake of omega acid-related supplement was restricted.

### Blood collection and biochemical analysis

Blood collections were performed via puncture of the antecubital vein, and samples were collected in 10-mL tubes containing heparin. Then, the tubes were centrifuged at 1,000 g for 15 min. The remaining plasma and plasma-TCA aliquot were stored in Eppendorf tubes at − 80 °C for later analysis. Plasma omega index was determined using a commercially available kit from OMEGA QUANT ASIA® (HIT #518, 17 Haengdang-dong, Seongdong-gu, Seoul 133–791, South Korea). All biochemicals were determined in duplicate and presented a mean variation of less than 5%. The HS-Omega-3 Index, which measured the levels of omega-3 fatty acids in red blood cells (RBCs), was assessed in red blood cells. This index specifically quantifies the percentage of EPA (eicosapentaenoic acid) and DHA (docosahexaenoic acid), two critical omega-3 fatty acids, in the total fatty acid content of RBC membranes. The method used for measuring the HS-Omega-3 Index generally involves the following steps. (1) Sample Collection (2) RBC Separation (3) Fatty Acid Analysis (4) Gas Chromatography (5) Calculation of the Index. The values of total saturated fatty acids (SFAs) and trans fatty acids (TFAs), Omega-3 (%), Omega-6 (%), Omega-7 (%), and Omega-9 (%) were included for evaluation of blood fatty acid composition. Insulin level (µU/mL), cholesterol (LDL, HDL), BUN/Cr, HbA1c_NGSP (%), Cystatin C (mg/L) were included for the evaluation of the endogenous factor.

### Statistical analysis

Data were expressed as mean and standard deviation (SD). All data were tested for distribution analysis using the Shapiro–Wilk test. Paired *t*-test or Wilcoxon’s signed-rank test were used to detect possible differences between the groups at the same time of blood collection and possible differences related to the time of blood collection (pre-seasonal, mid-seasonal, and post-seasonal). The level of significance was set at *p* < 0.05 in all analyses.

## Results

All data were expressed as mean with SD. Blood omega level was expressed as Percentage (%) of Total Fatty Acids. HS-omega-3 index was expressed as Percentage (%). total saturated fatty acids (SFAs) and trans fatty acids (TFAs) were expressed as Percentage (%) of Total Fatty Acids.

### Pre-seasonal data (baseline data, 1 month before season)

The mean values of blood omega-3 and omega-6 were 6.41 ± 1.5 and 34.3 ± 2.04, respectively. The mean value of omega-6/omega-3 was 5.71 ± 1.67. The HS-omega-3 index was analyzed as 7.01 ± 1.36. The values of total saturated fatty acids (SFAs) and trans fatty acids (TFAs) were reported as 42.80 ± 1.77 and 0.35 ± 0.12, respectively. Other data is presented in Table 1.

**Table 1:** Omega indices in each intervention

	Pre-seasonal	Mid-seasonal	Post-seasonal
HS_Omega-3 (%, mean [SD])	7.01 (1.36)	7.81 (1.05)	7.85 (1.29)
Omega-3 (%, mean [SD])	6.41 (1.50)	8.07 (1.20)	8.19 (1.60)
Omega-6 (%, mean [SD])	34.30 (2.04)	35.08 (2.24)	35.16 (1.52)
Omega-6/Omega-3 (mean [SD])	5.71 (1.67)	4.44 (0.72)	4.47 (0.96)
TFA (%, mean [SD])	0.35 (0.12)	0.76 (0.09)	0.81 (0.14)
SFA (%, mean [SD])	42.80 (1.77)	39.24 (1.10)	39.19 (1.16)
Omega-9 (%, mean [SD])	15.68 (1.19)	16.05 (1.78)	15.82 (0.91)
Omega-7 (%, mean [SD])	0.34 (0.21)	0.66 (0.28)	0.70 (0.19)

### Mid-seasonal data (5 month after the first game was started)

The mean values of blood omega-3 and omega-6 were 8.07 ± 1.20 and 35.80 ± 2.24, respectively. The mean value of omega-6/omega-3 was 4.44 ± 0.72. The HS-omega-3 index was analyzed as 7.81 ± 1.05. The values of SFA and TFA were reported as 39.24 ± 1.10 and 0.76 ± 0.09, respectively.

### Post-seasonal data (1 month after the last game was finalized)

The mean values of blood omega-3 and omega-6 were 8.19 ± 1.60 and 35.16 ± 1.52, respectively. The mean value of omega-6/omega-3 was 4.47 ± 0.96. The HS-omega-3 index was analyzed as 7.85 ± 1.29. The values of SFA and TFA were reported as 39.19 ± 1.16 and 0.81 ± 0.14, respectively.

### Difference in omega indices from baseline to mid-seasonal data

The mean differences in omega-3 and omega-6 were 1.66 (0.93, 2.40; *p* = 0.002) and 0.78 (− 0.30, 1.86; *p* = 0.146), respectively. The mean difference in omega-6/omega-3 was 1.27 (0.58, 1.96; *p* = 0.011). Furthermore, the mean difference in the HS-omega-3 index was 0.80 (0.15, 1.45; *p* = 0.017). The mean differences in total SFA and TFA were − 3.56 (− 4.55, − 2.56; *p* < 0.001) and 0.40 (0.34, 0.46; *p* < 0.001, respectively).

### Difference in omega indices from baseline to post-seasonal data

The mean differences in omega-3 and omega-6 were 1.77 (0.90, 2.55; *p* = 0.001) and 0.87 (− 0.35, 2.09; *p* = 0.153), respectively. The mean difference in omega-6/omega-3 was 1.24 (0.52, 1.96; *p* = 0.025). Furthermore, the mean difference in the HS-omega-3 index was 0.84 (0.19, 1.50; *p* = 0.014). The mean differences in total SFA and TFA were − 3.61 (− 4.65, − 2.57; *p* < 0.001) and 0.46 (0.37, 0.54; *p* < 0.001). Other data is presented in Table 2.

**Table 2:** Changes in omega indices from baseline to post-intervention

	Difference from baseline to mid*	*p*-value	Difference from baseline to post*	*p*-value
HS_Omega-3 (%, mean [SD])	0.80 (0.15, 1.45)	0.1956	0.84 (0.19, 1.50)	0.1619
Omega-3 (%, mean [SD])	1.66 (0.93, 2.40)	0.0016	1.77 (0.99, 2.55)	0.0015
Omega-6 (%, mean [SD])	0.78 (− 0.30, 1.86)	1	0.87 (− 0.35, 2.09)	1
Omega-6/Omega-3 (mean [SD])	1.27 (0.58, 1.96)	0.0114	1.24 (0.52, 1.96)	0.0205
TFA (%, mean [SD])	0.40 (0.34, 0.46)	< 0.0001	0.46 (0.37, 0.54)	< 0.0001
SFA (%, mean [SD])	−3.56 (− 4.55, − 2.56)	< 0.0001	−3.61 (− 4.65, − 2.57)	< 0.0001
Omega-9 (%, mean [SD])	0.37 (− 0.56, 1.30)	1	0.15 (− 0.39, 0.68)	1
Omega-7 (%, mean [SD])	0.32 (0.18, 0.45)	0.0009	0.35 (0.23, 0.48)	0.0001

*mid, mid-seasonal; post, post-seasonal. Data are expressed as mean (95% CI) or median (IQR). Paired *t*-test or Wilcoxon’s signed-rank test was used. For multiple comparisons, *p*-values were adjusted using the Bonferroni correction

### Endogenous factor-related blood test

No significant difference was observed in insulin, cholesterol (total, HDL, LDL) and BUN/Cr, HbA1c (NGSP), and cystatin C. Other data is presented in Table 3.

**Table 3:** Endogenous factor-related blood test

	Pre-seasonal	Post-seasonal	Difference from pre to post	*p*-value
Insulin (µU/mL, median [IQR])	6.75 [4.35, 8.48]	8.55 [6.97, 9.83]	−2.10[− 1.23, 4.43]	0.0929
Cholesterol_total (mg/dL, mean [SD])	197.15 (22.92)	192.95 (28.96)	4.20 [3.7, 12.1]	0.2822
Cholesterol_LDL (mg/dL, mean [SD])	124.40 (21.15)	119.10 (25.78)	5.30 (1.69, 12.29)	0.1291
Cholesterol_HDL (mg/dL, mean [SD])	66.85 (11.03)	62.65 (9.59)	4.20 (1.18, 7.22)	0.1334
BUN/Cr (mean [SD])	17.27 (3.54)	15.18 (2.30)	2.09 (0.36, 3.81)	0.3037
HbA1c_NGSP (%, mean [SD])	5.20 (0.23)	5.17 (0.21)	0.03 (0.03, 0.09)	0.3006
Cystatin_C (mg/dL, mean [SD])	0.81 (0.09)	0.78 (0.08)	0.03 (0.01, 0.05)	0.2428

## Discussion

This study showed that omega-3 enriched diet without the use of supplements can indeed increase the blood level of omega-3. Between the pre- and post-seasonal measurements, a mean difference in omega-3 of 1.77 (0.90, 2.55; *p* = 0.001) and omega-6 of 0.87 (− 0.35, 2.09; *p* = 0.153) was observed. Furthermore, the mean difference in the HS-omega-3 index was 0.84 (0.19, 1.50; *p* = 0.014). The mean differences in total SFA and TFA were − 3.61 (− 4.65, − 2.57; *p* < 0.001) and 0.46 (0.37, 0.54; *p* < 0.001).

There is growing evidence that n-3 supplementation reduces the production of proinflammatory cytokines and reactive oxygen species (ROS), which may indicate a direct association between recovery from intensive exercise and inflammation indicators [16]. Recent studies have demonstrated that dietary n-3 PUFA can increase physiological parameters such as blood flow during exercise, which are related to improved physical performance and oxygen utilization [17]. The nutrition plan for elite athletes of some professional teams includes supplements for boosting omega-3 [18–20]. However, there is little evidence that supplements increase blood levels of omega-3 FAs or change their composition. To the best of our knowledge, this is the first study to evaluate the biological assessment of the Omega-3 Status after omega-3 enriched dietary during an active seasonal performance on soccer players.

In active populations, chronic, low-grade inflammation may be associated with several overuse illnesses and disabilities. Concerns have been raised regarding prolonged oxidative damage causing inflammation. O3I has been linked to elevated muscle protein synthesis and decreased DOMS symptoms. Previous studies affirmed the significance of O3I monitoring, particularly among service members [21–25].

Even in the middle of the season, increased blood level of omega-3 can be maintained by following the n-3 PUFA-enriched dietary. The mean differences of omega-3 and omega-6 were 1.66 (0.93, 2.40; *p* = 0.002) and 0.78 (− 0.30, 1.86; *p* = 0.146), respectively. Omega-6/omega-3 had a mean difference of 1.27 (0.58, 1.96; *p* = 0.011). Furthermore, the mean difference of the HS-omega-3 index was 0.80 (0.15, 1.45; *p* = 0.017). Due to muscular overloading generated by high-degree exercise, soccer players must endure maximum muscle breakdown. The main goal of majority of soccer teams is to reduce exercise-induced muscle injury [25]. ARA, which is generally present in high concentrations in cell membranes, is altered by the incorporation of n-3 PUFAs into phospholipids. This results in the accumulation of EPA and DHA and decrease in AA levels. This may also potentially reduce the generation of ROS and inflammatory cytokines. N-3 PUFA and its primary bioactive FAs, EPA and DHA, are sources of antiinflammatory mediators, and their mechanisms of action have been identified [26]. We hypothesized that after a high-intensity exercise, n-3 PUFA may successfully alleviate muscle inflammation and enhance functional recovery [25]. One link between n-3 PUFA and muscle inflammation is the downregulation of proinflammatory cytokines such as TNF- and IL-6, which in turn leads to reduced AA and ROS generation and inflammatory response [16]. It remains unclear whether alteration of the omega-3 index and proportion of omega-6/omega-3 in blood is a clinically significant antiinflammatory factor; thus, exercise-related muscle damage can be reduced. Further clinical application should be investigated in the future.

## Conclusion

n-3 PUFA-enriched dietary supplementation might alter blood omega-3 indices in soccer players during the season.

## Data Availability

All data generated or analysed during this study are included in this published article and its supplementary information files.
